# Sphingosine‐1‐phosphate (S1P) receptors: Promising drug targets for treating bone‐related diseases

**DOI:** 10.1111/jcmm.15155

**Published:** 2020-03-10

**Authors:** Lincheng Zhang, Yutong Dong, Yiran Wang, Wenhui Hu, Shiwu Dong, Yueqi Chen

**Affiliations:** ^1^ Department of Biomedical Materials Science Third Military Medical University (Army Medical University) Chongqing China; ^2^ Battalion One of Basic Medical Sciences Third Military Medical University (Army Medical University) Chongqing China; ^3^ State Key Laboratory of Trauma, Burns and Combined Injury Third Military Medical University (Army Medical University) Chongqing China; ^4^ Department of Orthopedics Southwest Hospital Third Military Medical University (Army Medical University) Chongqing China

**Keywords:** cancer‐related bone metastasis, inflammatory osteolysis, osteoporosis, S1P receptors

## Abstract

Sphingosine‐1‐phosphate (S1P) is a natural bioactive lipid molecule and a common first or second messenger in the cardiovascular and immune systems. By binding with its receptors, S1P can serve as mediator of signalling during cell migration, differentiation, proliferation and apoptosis. Although the predominant role of S1P in bone regeneration has been noted in many studies, this role is not as well‐known as its roles in the cardiovascular and immune systems. In this review, we summarize previous research on the role of S1P receptors (S1PRs) in osteoblasts and osteoclasts. In addition, S1P is regarded as a bridge between bone resorption and formation, which brings hope to patients with bone‐related diseases. Finally, we discuss S1P and its receptors as therapeutic targets for treating osteoporosis, inflammatory osteolysis and bone metastasis based on the biological effects of S1P in osteoclastic/osteoblastic cells, immune cells and tumour cells.

## 1 INTRODUCTION

Bone is regarded as a highly dynamic tissue that constantly undergoes cycles of bone resorption and bone formation. The whole cycle is divided into three phases: (a) the interaction of osteoclast precursors (OCPs) and osteoblasts (OBs) on the bone surface, including OCP recruitment, RANKL‐RANK binding and adhesion to the bone surface; (b) the differentiation of osteoblast precursors (OBPs) is facilitated by various cytokines from mature osteoclasts; and (c) the apoptosis of osteoclasts (OCs), as well as mineralization of the bone matrix.^1^ During these OCPs and OBPs migrate to the bone surface, and various coupling factors, such as receptor activator of nuclear factor‐κ B ligand (RANKL), have a dramatic influence on the bone regeneration cycle. Currently, S1P has been recognized to participate in this remodelling cycle, suggesting its significance in bone pathology.

Sphingosine‐1‐phosphate (S1P) is a natural bioactive lipid molecule and a common first or second messenger in the cardiovascular and immune systems.^2^, ^3^ At present, research on the role of S1P in the regulation of cell migration, differentiation, proliferation and apoptosis is superficial.^2^ As a functional molecule that regulates numerous processes in the human body, S1P is increased in blood vessels and reduced in other tissues, thus creating an S1P gradient between blood and interstitial fluid. Moreover, this gradient determines the direction of cell migration, indicating that S1P is another chemokine that participates in chemotaxis in addition to C‐X‐C motif chemokine ligand 12 (CXCL12)/stromal cell‐derived factor 1 (SDF‐1). One of the causes of osteoporosis is chemokine‐mediated recruitment of osteoclasts into bone resorption sites, and inflammatory osteolysis, as well as bone metastasis, is due to the migration of inflammatory cells or tumour cells through chemokine gradients. In this context, studies on the S1P gradient are of great clinical value.

S1P is derived from ceramide, which is transformed into sphingosine primarily in vascular endothelial cells or circulating red blood cells/platelets. In addition, S1P is phosphorylated by sphingosine kinase 1/2 (SPHK1/2) and transported into blood vessels in different manners. While several transporters of S1P have previously been identified, spinster homologue 2 (SPNS2), a member of a large family of non‐ATP‐dependent organic ion transporters, has recently attracted much attention as an S1P transporter. Moreover, S1P is also exported from mast cells independently of their degranulation in a manner that is mediated by ATP‐dependent ABC transporters. Interestingly, the remaining S1P in different cells participates in several cellular behaviours via many other intracellular signalling pathways.^4^, ^5^ The difference between SPHK1 and SPHK2 is that SPHK1 mediates cytoplasmic S1P secretion, while SPHK2 phosphorylates S1P in cell nuclei to regulate histone acetylation.^6^ For the purpose of travelling through the blood, S1P must bind to high‐density lipoprotein (HDL) or albumin. Furthermore, over‐production of S1P can be inhibited to maintain a stable level. One mechanism is that intracellular S1P is degraded by sphingosine‐1‐phosphate lyase (SPL) or dephosphorylated by sphingosine‐1‐phosphate phosphohydrolase 1/2 (SPP1/2) after production, suggesting that S1P secretion is strictly controlled.^5^ As these enzymes are inactive in haemocytes, the S1P concentration in blood is much higher than that in other tissues, such as bone matrix. Another mechanism involves the lipid phosphohydrolase (LPP) family on the plasma membrane, which dephosphorylates extracellular S1P, resulting in reduced local extracellular S1P concentrations and increased sphingosine.^7^ The accumulated sphingosine takes part in other signal pathways or is converted back into S1P. Under normal circumstances, the generation and degradation of S1P maintain a dynamic balance. Disruption of this balance leads to dysfunction and diseases of many organs. In summary, S1P‐related enzymes may be future targets for treating bone disorders.

At present, five different S1P receptors (S1PR1‐5) have been identified and are encoded by *Edg1, 5, 3, 6* and *8*.^8^, ^9^ These receptors are G protein–coupled receptors and activate downstream signals after binding with S1P.^10^ Each receptor has a unique function and downstream signalling pathway based on its specific structure. S1PR1 is widely expressed in almost all kinds of cells and primarily couples to G_i/o_ proteins. S1PR2 and S1PR3 are less widely distributed and are coupled to G_12/13_, as well as to G_q_, G_s_ and G_i_. The expression of S1PR4 and S1PR5 is even more restricted and only detected in specific tissues. As the research on S1PRs is insufficient, especially in skeletal systems, there is an urgent need for more studies to investigate this field. Hence, this review will focus on how S1PR1‐3 influences bone metabolism and how to use these receptors as novel drug targets for bone‐related diseases. Because a multitude of S1PR agonists or antagonists are recognized and used in the laboratory to determine which S1PR is involved in certain functions, the development of our understanding of S1P has largely broadened.^10^ However, what is much more meaningful is that these activators or inhibitors can be used as drugs to treat bone diseases after commercial processing and extensive clinical trials. As osteolytic diseases such as osteoporosis and inflammatory osteolysis are partly caused by excessive numbers of osteoclasts, searching for a way to reverse excessive osteolysis without affecting bone turnover has incomparable superiority. However, raising this theoretical new therapeutic target to a practical level requires a large amount of effort. In this review, we will also focus on strategies for future research directions and drug targets.

## 2 THE FUNCTION OF S1P ON OSTEOCLASTS AND OSTEOBLASTS

As a dynamic metabolic system, the skeletal system undergoes degradation and renewal. Healthy people stably maintain this cycle, while patients with bone disorders suffer from disequilibrium of bone formation and resorption, leading to increased or decreased bone mass. As S1P has been discovered to play an essential role in bone metabolism, targeting S1PRs on osteoclastic and osteoblastic cells is a novel direction of scientific research. More importantly, S1P cooperates with RANKL to participate as a coupling factor in osteoclast‐osteoblast crosstalk. Thus, the clinical value of S1P involves the whole bone regeneration cycle.

### 2.1 The effect of S1P on osteoclasts

Osteoclasts are derived from the monocyte/macrophage lineage and fuse into multinuclear cells after stimulation with RANKL and macrophage colony‐stimulating factor (M‐CSF). These cells are responsible for bone matrix demineralization by secreting cathepsin K (CTSK) and HCl.^11^ Previous studies have discovered that S1P is responsible for the migration and differentiation of osteoclastic cells.

Some chemokines determine the direction of OCP migration, which play important role in bone resorption. S1PR1 and S1PR2, which were originally thought to be correlated with cell migration in lymphocytes, are more highly expressed than other S1PRs, indicating the impact of S1P on OCP migration.^12^ Based on a large number of experimental studies, S1P impacts OCP mobilization and recruitment. When the extracellular S1P concentration is low, S1P binds with S1PR1 to promote cell chemoattraction.^13^ Treatment with pertussis toxin, a Gi protein blocker, in a mouse model suggested that Gi and Rac were involved in S1PR1‐mediated chemoattraction. In contrast to this conclusion, a high level of extracellular S1P concentration results in S1PR2‐dominant regulation of OCP chemorepulsion. Many studies have shown that the G_12/13_/Rho signalling axis is downstream of S1PR2.^14^ Because OCP chemoattraction is enhanced when there are defects in S1PR2, a negative effect of S1PR2 signalling on the function of S1PR1 was determined. Interestingly, S1PR2^−/−^ mice showed a significant attenuation in osteolysis in vivo but not in vitro.^15^ This difference indicates that S1PR2 alone may not be sufficient to recruit osteoclast precursors. Ideas about the mechanisms of OCP chemotaxis suggest that S1PR1 is activated by S1P and rapidly internalizes in a high S1P environment and is transported back to the cell membrane in a low S1P environment. Therefore, S1PR2 is dominant when OCPs are circulating in the blood and is inhibited when OCPs are in the bone marrow. In addition, the binding of S1P to S1PR2 leads to reduced S1PR2 but increased S1PR1 expression, which forms a negative feedback loop. Moreover, osteoclast precursors, as well as bone marrow–derived macrophages/monocytes (BMMs), are stored in bone marrow and are mobilized to the blood when the expression of S1PR1 reaches a level that can be activated by low S1P concentrations.

Based on previous studies, RANKL and M‐CSF play important roles in osteoclast differentiation. In addition, S1P participates in OCP differentiation in an indirect manner. Usually, S1P impacts this process by regulating RANKL expression or its downstream signalling pathway. The existence of SPHK1, which is responsible for S1P production, sharply reduces osteoclastogenesis in BMM cultures by blocking p38 MAPK, c‐Fos and NFATc1 and augmenting ERK.^16^ Intriguingly, coculture of BMMs with osteoblasts had the opposite effect and increased RANKL expression.^17^ The difference between these two culture systems subtly revealed that intracellular S1P in OCPs suppresses OCP maturation and boosts RANKL expression by osteoblasts after secretion from the cells.^18^ Moreover, enhanced RANKL expression induces a dramatic increase in BMM differentiation. Previous studies found two different mechanisms for bone resorption: (a) chemorepulsion of osteoclast precursors through the S1P gradient and (b) an increase in RANKL due to osteoclast‐secreted S1P. The S1P gradient between the blood and bone matrix, as well as S1PR2 on OCPs, is more important for bone resorption, suggesting that the increased number of osteoclasts plays a more superior role than enhanced osteoclast maturity.^19^ However, this does not mean that the differential effect of RANKL is unimportant. In contrast, monocytes are unable to fuse with mature osteoclasts in the absence of RANKL. After fusing into multinuclear cells, osteoclasts must adhere to the bone surface to secrete H^+^, cathepsin K and matrix metalloproteinases (MMPs). The markers of osteoclast formation and activation, including NFATc1, CTSK, acid phosphatase 5 (ACP5), osteoclast‐associated receptor (OSCAR), dendritic cell–specific transmembrane protein (DC‐STAMP) and osteoclastic cell–specific transmembrane protein (OC‐STAMP), sharply increase after S1PR2 stimulation.^20^

### 2.2 The effect of S1P on osteoblasts

Osteoblasts, unlike osteoclasts, are derived from mesenchymal stem cells (MSCs).^21^ These cells generally remain on the bone surface and develop into osteocytes in the bone matrix. Collagen and other substances are secreted by osteoblasts for bone mineralization and formation, which is a key step in bone remodelling. Because osteoblasts are responsible for bone mineralization, their recruitment, differentiation and proliferation are fundamental for bone formation.

Similar to OCPs, S1P regulates OBP mobilization and recruitment through S1PR1/S1PR2 downstream signals. S1PR1 stimulates the JAK/STAT signalling axis, while S1PR2 activates the FAK/PI3K/AKT signalling axis, both of which promote MSC migration.^22^ S1PR2 mediates chemorepulsion of OBPs, while OBP chemoattraction is modulated by S1PR1.^2^, ^23^, ^24^ Further studies also showed that the JAK/STAT and FAK/PI3K/AKT signalling axes were independent and had no crosstalk.^24^

In addition to osteoblast location, S1P is also crucial for OBP differentiation. S1PR1‐3 is largely expressed in osteoblastic cells. In contrast to the decrease in S1PR1 and S1PR2 expression, S1PR3 expression increases sharply during OBP differentiation. Moreover, when S1PR3 is knocked out, bone matrix mineralization is not impacted by S1P, which means that S1PR3 regulates osteoblastogenesis. Other studies also found that S1PR1 and S1PR2 participate in osteoblastogenesis despite their decreased expression during differentiation. Bone morphogenetic protein 2 (BMP2), a crucial protein for OBP differentiation, is the target for S1PR1/2 downstream signalling pathways. The phosphorylation of Smad1/5/8, as well as ERK1/2, and the expression of Runx2 increase dramatically after activation of S1PR1 and, to a lesser extent, S1PR2.^25^ However, recent studies discovered that the PI3K/AKT/GSK‐3β/β‐catenin and RhoA/ROCK/Smad1/5/8/Runx2/ALP signalling axes were non‐BMP2‐dependent pathways for osteoblastogenesis that signalled through S1PR1 and S1PR2, respectively. RhoA was also responsible for Smad 6/7 phosphorylation, which inhibits Smad1/5/8 activation and represents negative feedback regulation.^26^ In contrast, S1P enhances S1PR1 and S1PR2 expression, which is positive feedback regulation. Overall, S1PR1‐3 promotes OBP differentiation via a regulatory signalling network. In this network, S1PR3 directly regulates osteoblastogenesis, while the differential effect of S1PR1/2 is less powerful than that of S1PR3.

Since mature osteoblasts still undergo mitosis, osteoblast proliferation and survival are crucial for osteogenesis. Various studies have found that S1P facilitates osteoblast proliferation and survival.^27^, ^28^, ^29^, ^30^ Initially, only p42/44 MAPK and G_i_ were identified as downstream signalling pathways of S1P in the proliferation process.^31^ Later, studies discovered that PKCs are increased by S1P, and protein kinase C α (PKCα) was considered a downstream signal of S1PR1.^7^ Some researchers even held the idea that PKC cooperates with MAPK to exert its proliferation effect.^31^ However, a group of scientists found that intracellular [Ca^2+^] but not PKC was required for S1P‐induced p42/44 MAPK activation, indicating that PKC exerts a proliferative effect without activating MAPK.^32^ Calcium is found mainly in bone, and intracellular [Ca^2+^] plays an essential role in bone metabolism and metastasis.^33^, ^34^, ^35^ Therefore, the involvement of the calcium signalling pathway in S1P‐induced osteoblast proliferation significantly improves our understanding of the function of [Ca^2+^] in bone homeostasis. Furthermore, pertussis toxin (a Gi inhibitor), LY294002 or wortmannin (PI3K inhibitors) and calphostin C (a PKC inhibitor) treatment were used on osteoblasts to determine which downstream signal of S1PR impacts osteoblast apoptosis. The results revealed, as expected, that G_i_ proteins and PI3K are the downstream signals of S1P for anti‐apoptotic effects in osteoblasts.^36^ Research on the function of S1P receptors in osteoblast proliferation and survival is still far from sufficient, and further studies will be of great clinical value for bone‐related diseases. Despite limited evidence, mechanical loading was found to stimulate S1P production in osteocytes via up‐regulating SPHK1 and down‐regulating SPP, SPL and SPNS2. The mechanism occurred by the osteocyte network translating the force they have experience into a biological response, such as bone regeneration.^37^

### 2.3 The effect of S1P on the crosstalk between osteoblasts and osteoclasts

**Figure 1:**
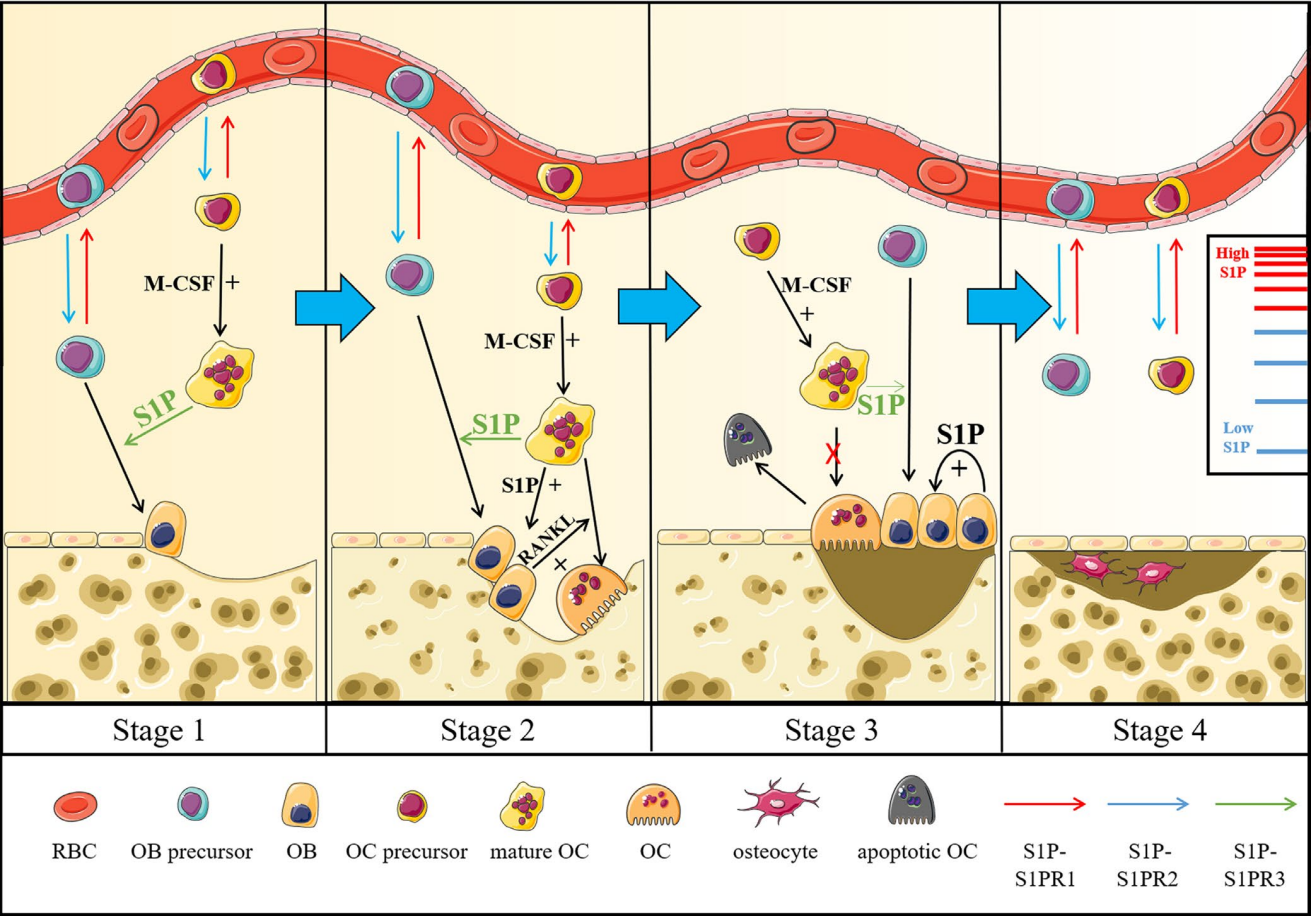
S1P plays an essential role in bone turnover and osteoporosis. One of the early signs of osteoporosis is obstacles to bone turnover, and the whole process of bone turnover can be divided into four stages. In the first stage, OCPs and OBPs recruit to bone marrow via S1PR2 signal pathway (these cells are mobilized into blood through S1PR1 signalling). And osteoclastic cell‐secreted S1P can stimulate the differentiation of osteoblasts. In the second stage, RANKL, cooperating with S1P, acts as a coupling factor for osteoclast‐osteoblast crosstalk, which facilitates bone turnover and maintains bone haemostasis. The differentiation of osteoblasts and the expression of RANKL are induced by S1P (released from osteoclastic cells), while the dissolution of bone matrix will release TGF‐β. In the third stage, the rapid proliferation of osteoblasts (induced by S1P in an autocrine manner) and the apoptosis of osteoclasts lead to the increase of OB/OC ratio, which eventually refill the dissolved bone matrix. In the last stage, the formation of new bone is complete and bone turnover will resume from the first stage

As previously stated, S1P is crucial for the differentiation, proliferation and migration of both osteoclasts and osteoblasts. For cell migration of osteoclasts and osteoblasts, the same S1PR (S1PR1/2) may exert the same effect. Therefore, by selectively activating or blocking specific S1PRs on specific cells or by impacting S1PRs on different cells to different degrees we can selectively regulate the migration of OCPs or OBPs individually. In addition, it has been gradually accepted that osteoclasts and osteoblasts are not completely antagonistic cells. In contrast, they are tightly coupled and interact with each other to maintain homeostasis and bone turnover (Figure 1). Currently, S1P is generally regarded as another coupling factor that mediates bone regeneration. More specifically, S1P depends on stimulating certain S1PRs or facilitating the downstream signalling of other coupling factors, such as RANKL, to perform osteoclast‐osteoblast crosstalk. Since S1P is mainly secreted by osteoclasts in the bone matrix, osteoclasts may play an important role in regulating osteoblast migration, differentiation and proliferation (Figure 1). Moreover, S1P enhances the expression of RANKL in osteoblasts, which promotes osteoclast differentiation and controls osteolysis (Figure 1). Thus, S1P, as a coupling factor between osteoclasts and osteoblasts, stimulates the mutual promotion and balance of osteolysis and osteogenesis. A recent study found that overexpression of intracellular S1P in BMMs attenuated osteoclastogenesis, while it stimulated RANKL expression to promote osteoclast differentiation after secretion into the bone matrix.^16^ Therefore, inhibiting the release of S1P from osteoclastic cells while increasing S1P production in osteoclastic/osteoblastic cells may greatly disrupt osteoclastogenesis without affecting bone turnover. Although some studies have already been carried out in this field, there is a strong need to perform further studies (Table 1).

**Table 1:** Biological functions of S1P in skeleton system and its downstream signal axis

Biological effects of S1P in skeleton system			
Cell	Effect	Signal pathway	References
OCP	Migration	S1PR1/G_i_/Rac/chemoattraction	12, 13
		S1PR2/G_12/13_/Rho/chemorepulsion	13, 14
	Differentiation	S1P/COX2 and mPGES1/PGE2/RANKL/RANK	16
OBP	Migration	S1PR1/JAK/STAT/chemoattraction	22
		S1PR2/FAK/PI3K/AKT/chemorepulsion	22
	Differentiation	S1PR1/PI3K/AKT/GSK‐3β/β‐catenin	25, 26
		S1PR2/RhoA/ROCK/Smad1/5/8/Runx2 and ALP	25, 26
		SPHK1/S1P/S1PR3/Runx2 and ALP	39
OB	Proliferation	Gi/p42/44 MAPK	31
		Intracellular [Ca^2+^]/p42/44 MAPK	33, 34, 35
		PKCα	7
	Survival	Gi/PI3K	38
	IL‐6 synthesis	p42/p44 MAPK	40
OC	Activation	S1PR2/RANKL/NFATc1, CTSK, ACP5, OSCAR, DC‐STAMP and OC‐STAMP secretion	20

## 3 THE EFFECT OF S1P ON OSTEOPOROSIS

Osteoporosis is currently the most common osteolytic disease among elderly individuals and is typically characterized by a decrease in bone mass/density and bone strength. Osteoporosis is prone to complications such as long bone fracture. Osteoporosis occurs for many reasons, but it is essentially a disorder of bone formation and resorption.^41^ Because of the widespread prevalence of osteoporosis, studies searching for potential therapeutic targets are relatively old.

The traditional drug for osteoporosis is bisphosphonate, which is also used in the treatment of Paget's disease, multiple myeloma and bone metastasis.^42^ It is a synthetic analogue of pyrophosphate combined with hydroxyapatite, and its anti‐osteolytic effect is due to inhibition of bone resorption as well as promotion of osteoclast apoptosis, which may lead to severe side effects such as gastrointestinal, renal and ocular toxicities and osteonecrosis of the jaw (ONJ) after long‐term treatment.^43^ At present, bisphosphonate is still the primary drug for osteoporosis, which means that exploring a novel treatment to reduce the side effects still has clinical value. Based on this purpose, we found a new drug called denosumab, a RANKL monoclonal antibody, that inhibits osteoclast differentiation with a much less negative effect than that of bisphosphonate. However, long‐term use of denosumab leads to low bone turnover, which means that patients with severe bone regeneration problems should avoid using it.^44^ Currently, a humanized monoclonal sclerostin antibody called romosozumab promotes the Wnt signalling pathway in osteoblastic cells, which increases bone formation and mineral density.^45^ Although it has significant advantages over other osteoporosis drugs, it still leads to a much larger number of osteoblasts than osteoclasts and will weaken the coupling between these two cell lineages.^46^ Compared with the effects of these drugs, treatments that work by modulating S1P can influence both osteoclasts and osteoblasts at the same time. Osteoclasts will not be over‐inhibited when treating osteoporosis. Therefore, S1P, acting as a crucial factor to enhance bone regeneration, can be used to treat osteoporosis by restoring the dynamic balance between osteolysis and osteogenesis.

By modulating S1PRs on osteoclasts or osteoblasts, their effects against osteoporosis were discovered. For example, S1PR1 agonists or S1PR2 antagonists decrease osteoclasts in the bone matrix, while S1PR3‐specific agonists are responsible for osteoblast differentiation. These agonists or antagonists serve as effective and valuable drugs for osteoporosis. However, the most promising S1P‐related therapeutic targets for osteoporosis at present are the enzymes involved in S1P metabolism. Weske et al provided a potential anabolic therapy for bone loss by targeting S1P lyase.^47^ In addition, calcitonin (CT) was proven to promote *Spns2* (the gene determining the expression of SPNS2) transcription, which enhanced the release of S1P from osteoclasts.^48^ Similarly, the formation of S1P can be modulated by treatments targeting SPHK. Using SKi (10 μmol/L), a SPHK inhibitor, in hOB assays further confirmed this hypothesis.^49^ Because these enzymes have limited distribution and their activation can be easily controlled, controlling the S1P concentration by regulating enzyme activation has tissue specificity, and they have great advantages over directly regulating S1PRs.

Moreover, S1P also regulates the function of hormones associated with osteoporosis. Calcitonin (CT) was found to be involved in bone loss in a non‐canonical manner, which abolished the secretion of S1P from osteoclastic cells to inhibit osteoblast differentiation.^48^ Oestrogen acts as an important mediator in regulating bone matrix metabolism, which also participates in the activation of an intracellular network composed of many cytoplasmic and nuclear mediators. Additionally, some oestrogen effects can also be mediated by sphingolipids. Furthermore, oestrogen activates S1P receptors (S1PRs) and induces growth factor receptor transactivation.^30^, ^50^ 17‐β‐oestradiol (E2), one of the three major types of endogenous oestrogens, exerts a direct osteogenic effect or regulates osteogenesis via the S1PR1/SPHK/S1P signalling axis (the concentration of E2 used was 10 nmol/L).^30^ Another hormone that is highly correlated with S1P is glucocorticoid. In addition to their anti‐inflammatory and immunosuppressive functions, glucocorticoids have also been widely used in clinical treatment and in rescuing emergency patients in the medical community. However, the negative effect on osteoblast survival is a major concern when considering glucocorticoid use. A recent experiment using K6PC‐5, an activator of SPHK1, in MC3T3‐E1 osteoblastic cells successfully reversed Dex‐induced apoptosis, indicating that S1P greatly alleviates the side effects of glucocorticoids on bone.^38^ Overall, excessive secretion or the use of these osteolytic hormones leads to decreased osteoblasts or increased osteoclasts, and regulating S1P is a novel idea to treat osteoporosis. However, the links between oestrogen signalling and activation of sphingosine kinase axis in bone cells are unclear and further trials are needed to determine whether the treatment can be used clinically. It has also been shown that sphingosine kinase and S1P receptors participate in oestrogen‐mediated EGF receptor transactivation. Moreover, some functional proteins cooperate with S1P to mediate bone metabolism, including epidermal growth factor (EGF) and bone morphogenetic protein 6 (BMP6).^51^ In previous studies, EGF was found to trigger osteoblast proliferation by increasing intracellular S1P concentrations, while OC‐secreted BMP6, together with S1P, modulated osteoblastogenesis and mineralization.^52^, ^53^ In addition, a current study using CTSK^−/−^ osteoclasts resulted in enhanced SPHK1, demonstrating that deletion of CTSK enhances bone formation in vivo by increasing the generation of osteoclast‐derived S1P.^54^ Furthermore, because crosstalk between the apoptosis molecules Fas and S1PR1 was found in the osteoclasts of rheumatoid arthritis (RA) mice, S1P was thought to be correlated with osteoclast apoptosis.^32^, ^36^, ^55^ Although studies on S1P‐induced osteolysis or osteogenesis have focused on many molecules, further investigation is still needed to explore their appropriate clinical dosage and possible side effects.

## 4 THE EFFECT OF S1P ON INFLAMMATORY OSTEOLYSIS

Inflammatory osteolysis is a common but severe disease, causing millions of people around the world to suffer from bone density loss. It is the result of bone infection and is characterized by overactivated osteoclasts and an imbalanced bone remodelling cycle.^56^ Currently, surgical treatment is the only efficient method to address inflammatory osteolysis, but it may cause serious prognostic difficulties and mobility problems for patients. Therefore, finding a non‐surgical treatment has great clinical value. Based on previous studies, there is a connection between S1P and inflammatory reactions, indicating that S1P may be a future target for inflammatory osteolysis.

**Figure 2:**
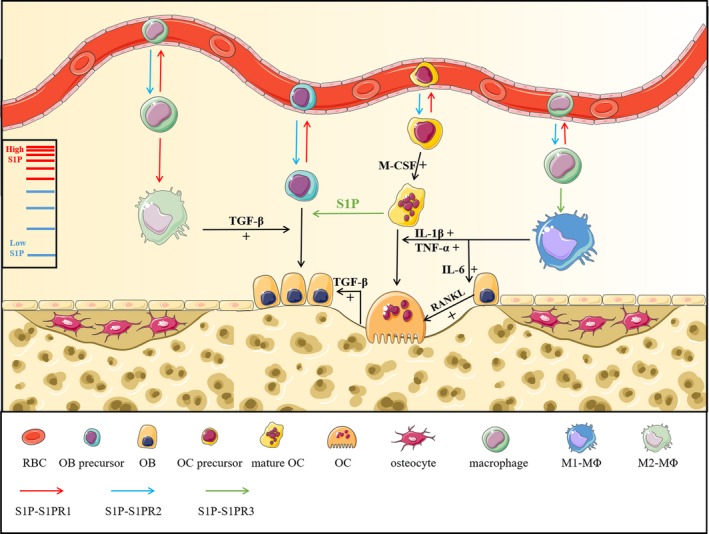
As one of inflammatory mediators, S1P serves as a mediator for inflammatory osteolysis. Excessive osteoclast proliferation and destruction of bone homeostasis are the main characteristics of inflammatory osteolysis, and macrophages are found to participate in this process. Macrophages can be recruited into inflamed sites of bone marrow, and S1P acts as a promoter for the polarization of M0‐MΦs into M1‐MΦs via S1PR3 signal pathway. Besides, the release of TNF‐α, IL‐1β and IL‐6 from M1‐MΦs can interact with S1P to participate in the regulation of bone turnover. TNF‐α and IL‐1β exert direct promotional effect on osteoclast differentiation, while IL‐6 elevates RANKL expression to boost osteoclastogenesis. In addition, the polarization of M2‐MΦs can be stimulated by S1PR1 signal pathway, which exerts osteogenic effect by secreting TGF‐β

When G^−^ bacteria infect bone tissue, their cell wall component LPS is released to the bone matrix, which activates macrophages by binding to Toll‐like receptor 4 (TLR4). Activated macrophages, called M1 macrophages, release a variety of pro‐inflammatory cytokines, such as tumour necrosis factor‐α (TNF‐α), interleukin‐1β (IL‐1β) and interleukin‐6 (IL‐6), and enhance the expression of inducible NO synthase (iNOS), C‐C motif chemokine receptor 7 (CCR7) and CD86.^57^ TNF‐α and IL‐1β directly promote osteoclastogenesis, while IL‐6 only stimulates osteolysis by stimulating RANKL expression on osteoblasts (Figure 2).^56^, ^57^ More importantly, M1 macrophages differentiate into osteoclasts after binding to RANKL, indicating that IL‐6 also promotes osteoclast differentiation from M1 macrophages by increasing RANKL.^58^ Recently, a group of scientists discovered that M1 macrophages also stimulate RANKL expression via the SPHK1/S1PR1/RANKL signalling axis, forming a positive feedback loop.^57^ Surprisingly, other experiments showed that S1P increased COX‐2, iNOS, prostaglandin E2 (PGE2), IL‐1β and TNF‐α in several cell lineages, including murine peritoneal macrophages.^59^, ^60^, ^61^, ^62^ These findings indicate that S1P may also play an essential role in LPS‐mediated inflammatory osteolysis. Many macrophage lineages express S1PR1‐3, and their pro‐/anti‐inflammatory functions have not been completely discovered. Heo *et al* examined the expression of pro‐inflammatory cytokines induced by LPS. Not only COX‐2 and iNOS but also IL‐1β, IL‐6 and TNF‐α were inhibited after treatment with the S1PR3 inhibitor TY52156, demonstrating the pro‐inflammatory effect of S1PR3 (Figure 2).^63^ Moreover, in a previous study by Keul et al, S1PR3^−/−^ macrophages did not migrate towards S1P, suggesting that S1PR3 participates in macrophage chemoattraction.^64^ Thus, S1PR3 signalling is responsible for both macrophage mobilization and M1 macrophage polarization (pro‐inflammatory effect). A recent study discovered that S1PR2/3 stimulates M1 polarization via the G_(α)i/o_/PI3K/JNK signalling axis in liver inflammation. However, whether this signalling pathway is involved in inflammatory osteolysis is still unclear.^65^ Another study carried out by Hughes et al identified that S1PR1 on macrophages exerts an anti‐inflammatory effect.^66^ Macrophages that produce IL‐4 and IL‐13 as anti‐inflammatory cytokines are M2 macrophages. Further study using VPC44116, an S1PR1‐specific antagonist, showed decreased arginase I (Arg I) activity (a marker of M2 macrophages) and increased iNOS activity (a marker of M1 macrophages), indicating that S1PR1 signalling is responsible for M2 macrophage polarization.^66^ Intriguingly, in ovariectomized (OVX) mice, M2 macrophages differentiated into osteoclasts, and an increased M1/M2 ratio was also discovered, suggesting a connection between oestrogen deficiency, osteolysis and M1 macrophage polarization.^67^ As the direction of macrophage polarization is determined by the expression of certain S1PRs or environmental S1P concentrations, it is possible to transform M1 macrophages into M2 macrophages in the bone matrix. If this idea can be used in clinical treatment, we would be able to reverse excess osteoclasts or even control the progression of inflammatory osteolysis. In addition, M1 and M2 macrophages exert completely opposite effects, and so identifying how S1PR1 and S1PR3 affect macrophages will help in regulating the M1/M2 ratio. We also hypothesize that the same S1PRs (S1PR1 or S1PR3) may exert different functions in different tissues during inflammatory reactions, and so the function of S1PR1 and S1PR3 may not strictly follow the conclusions above. For example, a recent study found that S1PR1 signalling up‐regulates IL‐6 expression in primary mouse macrophages by activating JAK2.^68^ In addition to S1PR1 and S1PR3, S1PR2 also induces its anti‐inflammatory effect by inhibiting macrophage recruitment.^69^ Moreover, using an anti‐IL‐6 receptor antibody efficiently down‐regulated S1PR2 in a collagen‐induced arthritis (CIA) model, causing severe osteolytic disease, which further suggests the anti‐inflammatory effect of S1PR2.^70^ Thus, S1PR1‐3 on macrophages is potential targets for inflammatory osteolysis, and regulating the secretion of pro‐/anti‐inflammatory cytokines may be a future direction for clinical research. However, there is still a lack of evidence determining their detailed downstream signalling pathways.

Macrophage‐produced cytokines not only directly or indirectly impact the formation and activation of osteoclastic cells but also act as inflammatory mediators or chemokines to recruit lymphocytes to execute adaptive immunity reactions. Surprisingly, the S1P gradient between the blood/lymph and other organs is also responsible for lymphocyte trafficking from the thymus to inflamed sites and is mediated by S1PR1 signalling.^2^, ^71^, ^72^, ^73^ If the inflamed site is bone, recruited lymphocytes directly eliminate pathogenic bacteria and control the progression of inflammatory osteolysis. In addition, lymphocytes are currently thought to be the only cell lineage to express S1PR4. A previous study showed that S1PR4 was associated with neutrophil migration during inflammation.^71^ Therefore, S1PR4 may be a new drug target for inflammatory osteolysis. In addition, inflammation‐induced lymphangiogenesis is widely accepted and is beneficial for lymphocytes and tumour cells. Recently, S1PR1 on HLECs was found to stimulate TNF‐α and IL‐1β secretion via the NF‐κB signalling pathway, and TNF‐α and IL‐1β are essential for HLEC proliferation, migration and lymphangiogenesis.^74^ Although inflammation is a hot spot in basic medical research, there are still relatively few studies on the skeleton system. Based on the results shown in other tissues, lymphocyte recruitment and cytokine secretion may be potential therapeutic targets for inflammatory osteolysis.

Inhibiting inflammatory osteolysis through the intervention of S1P and lymphocyte recruitment has great advantages compared with those of formal surgical methods (mainly by removal of a large segment of infected bone tissue). Thus, there is a strong need to investigate the mechanisms by which S1P affects inflammation and lymphocyte recruitment.

## 5 THE EFFECT OF S1P ON CANCER‐RELATED BONE METASTASIS

Cancer is regarded as the top killer among patients worldwide and is characterized by uncontrolled growth, infiltration and metastasis. Tumour metastasis is the leading cause of cancer death, and bone is the third most common site for tumour metastasis after the lung and liver.^75^ According to previous studies, breast cancer (BCa) and lung cancer (LCa) usually metastasize to bone, causing severe pain and osteolysis. In addition, prostate cancer (PCa)–related bone metastasis always results in bone fracture.

**Figure 3:**
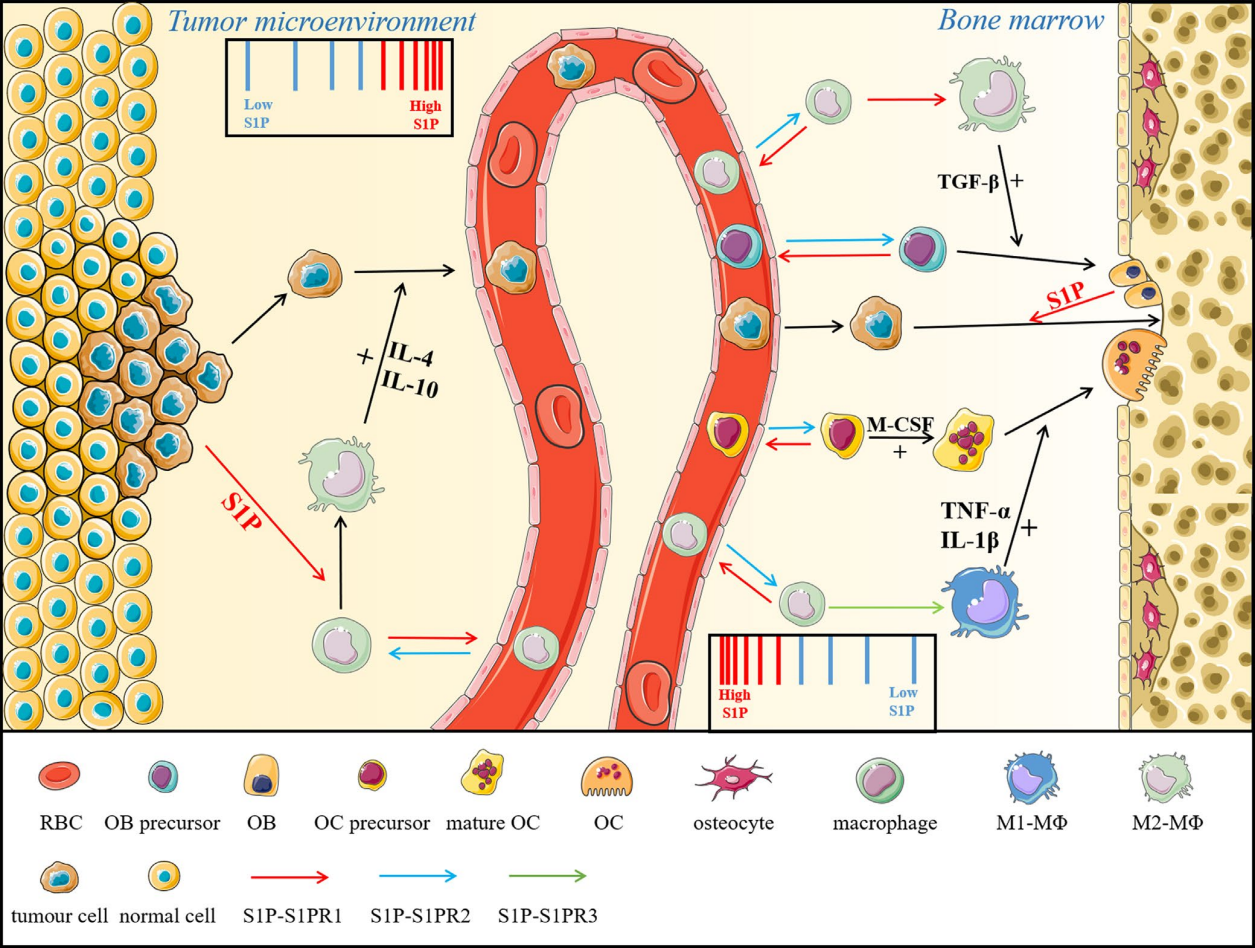
S1P participates in regulating cancer‐related bone metastasis in bone marrow and tumour microenvironment. In tumour microenvironment, apoptotic tumour cell‐released S1P is responsible for the polarization of M2‐MΦs in a S1PR1‐dependent manner. IL‐4 and IL‐10 are formed by M2 macrophages, which are crucial for tumour cell evasion, whereas in bone marrow, metastasized tumour cells colonized to bone surface, and S1P (secreted by osteoblasts) promotes their proliferation depending on S1PR1 signalling

Recently, various studies have found a link between S1P and bone metastasis, providing us with a novel drug target for bone metastasis. At the early stage of metastasis, S1P participates in the metabolism and migration of primary tumour cells in the tumour microenvironment by cooperating with tumour‐associated macrophages (TAMs). TAMs act to eliminate tumour cells and are recruited to the tumour microenvironment via S1P‐mediated ‘find me signals’.^76^ S1P in the tumour microenvironment is secreted and released by apoptotic tumour cells due to SPHK1 activation.^76^ Recruited monocytes undergo macrophage polarization (differentiation into M2 macrophages) after stimulation with S1P (Figure 3).^77^, ^78^ Among the cytokines formed by M2 macrophages are IL‐4 and IL‐10, which promote tumour evasion and chemotherapy resistance (Figure 3).^79^ Moreover, M2 macrophages in the inflammatory tumour microenvironment also release PGE2 to modulate angiogenesis, further promoting tumour progression and metastasis.^80^ Because S1PR3 downstream signalling promotes M2 macrophage polarization, using an S1PR3 antagonist in the tumour microenvironment may block the formation of M2 macrophages and fundamentally reduce the possibility of bone metastasis. Furthermore, transforming M2 macrophages into M1 macrophages may be a future strategy for controlling tumour spread. However, there are relatively few studies about how to transform M2 macrophages into M1 macrophages, and the changes caused by M1 macrophage‐secreted cytokines cannot be estimated. Therefore, the effect of M1 macrophage‐secreted cytokines on the tumour microenvironment is of great academic and clinical value. In addition, primary tumours are difficult to cure because of immune tolerance, which makes it difficult to achieve the expected therapeutic effect by immunological methods. Therefore, targeting M2 macrophages to control bone metastasis will be possible only if we find a way to reduce immune tolerance. In the last few years, various studies have focused on the role of T_reg_ cells in immune tolerance. Tumour‐specific T_reg_ cells were thought to egress from bone marrow to tumour tissue in breast cancer patients by the stimulation of S1PR1, according to Rathinasamy et al This group also found that S1PR1 expression in T_reg_ cells was induced after binding with antigen.^81^ Thus, S1P is correlated with T_reg_ cell–mediated immune tolerance. However, a brain‐specific mechanism by which tumours escape immunosurveillance though the loss of S1PR1 on T cells was discovered.^27^ Thus, the exact mechanism by which S1P induces immune tolerance is not obvious, and it is unwise to blindly suppress S1PR1 on Τ cells or change the environmental S1P concentration.

Migration of tumour cells through blood and lymph vessels is a key step in tumour metastasis, and S1P seems to participate in this process via various mechanisms. On the one hand, the function of cytotoxic T cells is attenuated after binding to circulating S1P, promoting immune tolerance and preventing the elimination of tumour cells.^82^ On the other hand, S1P secreted by lymphatic or vascular endothelial cells inhibits breast cancer metastasis‐suppressor 1 (BRMS1) after binding to S1PR2 on tumour cells.^83^ Furthermore, lymphangiogenesis and angiogenesis are both essential elements in tumour metastasis, and it is the main reason why tumour metastasis has a close interaction with inflammation. As mentioned above, stimulating S1PR1 on HLECs promotes lymphangiogenesis via the secretion of TNF‐α and IL‐1β, which are known pro‐inflammatory cytokines. In addition, a previous study investigated another mechanism of S1P‐induced lymphangiogenesis, in which S1P was produced by tumour cells.^84^ As these results were not determined in the cancer lineages that usually metastasize to bone, we are not sure whether these mechanisms are involved in bone metastasis. Thus, determining the mechanisms in PCa/BCa cell lineages is a future strategy for treating bone metastasis.

Finally, OB/OC‐secreted S1P recruits certain tumour cells by enhancing the connection between tumour cells and the osteoclastic/osteoblastic niche, which is called bone colonization (Figure 3). An early experiment using several PCa cell lines co‐cultured with osteoblastic cell lines (mainly MC3T3 cells) showed increased proliferation and resistance to standard therapy. This effect was due to OB‐secreted S1P binding to S1PR1 on PCa cells and forming a positive feedback loop by promoting S1PR1 expression.^39^ Because PCa is involved osteogenic bone metastasis, osteoblastic niches may act as S1P reservoirs for tumour cell proliferation. Although experimental records have not shown whether osteoclastic niches act as S1P reservoirs for bone metastasis in BCa/LCa, we make this assumption based on the consensus that osteoclasts are better at secreting S1P than osteoblasts and that BCa/LCa are associated with osteolytic bone metastasis. More importantly, regulating S1P‐related enzymes may also serve our purpose. However, we discovered that targeting a single receptor–ligand pro‐metastatic axis to treat tumour metastasis cannot effectively inhibit the spread of cancer in the body.^84^ Thus, there is a strong need for more research to find other therapeutic targets, and a therapy that combines different mechanisms of bone metastasis is also a future direction for clinical treatment.

## 6 THE CLINICAL DEVELOPMENT OF DRUGS TARGETING S1P RECEPTORS IN TREATING BONE‐RELATED DISEASES

Due to the predominant role of S1P receptors in bone‐related diseases have been widely noted, it is necessary to develop and test drugs that target S1P receptors in patients with bone‐related diseases. Among the several kinds of different drugs targeting one or more S1P receptors, fingolimod, ponesimod, siponimod, ozanimod and other related drugs are participated in clinical application and further development. Fingolimod (FTY720) acts as an agonist of the S1P receptor (S1PR). It could induce internalization and subsequent degradation of the receptor and consequently render lymphocytes to physiological S1P stimulation upon binding to the S1PR expressed on lymphocytes.^85^ In addition to its immunology modulatory effects in MS, fingolimod may have a beneficial effect on bone mass loss in female MS patients.^57^ Furthermore, fingolimod has been reported that it dramatically prevented bone loss in vivo via inhibiting RANKL‐induced osteoclastogenesis in periodontitis model.^57^ Additionally, fingolimod was shown that it could also attenuate cancer‐induced spontaneous pain.^86^ Moreover, ponesimod is a selective S1P1R regulator to participate in the dose‐dependent sequestration of lymphocytes in lymphoid organs.^87^ It has been noted that ponesimod acts as the potential treatment for MS and other immune‐mediated diseases, so that the treatment for bone‐related diseases through immunoregulatory mechanism is also a potential choice.^87^ BAF312 (siponimod), a dual agonist at S1PR1 and S1PR5, which is currently undergoing clinical trials for the treatment of secondary progressive multiple sclerosis (MS).^88^ Besides reducing inflammation by sequestering lymphocytes in lymphoid tissues, BAF312 could also cross the blood‐brain barrier and binds its receptors on many kinds of cells.^88^ Similarly, ozanimod (RPC1063) is a specific and potent small molecule modulator of the S1PR1 and S1PR5, which has shown therapeutic effect on relapsing MS and ulcerative colitis.^89^ In many other autoimmune diseases, ozanimod (RPC1063) also plays dramatically essential role in the treatment for them.^89^ Thus, many bone‐related diseases could also be regarded as the autoimmune diseases so as to be treated effectively. Above all these, these different drugs targeting one or more S1P receptors have the potential effect in the treatment for bone‐related diseases.

## 7 CONCLUSION

Although scientific research on the relationship between S1P and bone biology is much more restricted than that of the cardiovascular and immune systems, S1P still has tremendous clinical value in bone pathology and will become a promising target for the treatment of many bone‐related disorders, such as osteoporosis, inflammatory osteolysis and bone metastasis.^90^, ^91^ A better understanding of S1P in osteoclastic/osteoblastic cells, as well as related signal pathways, is in great demand.^55^ In addition to the role of S1P as a first or second messenger that regulates cell migration, differentiation, proliferation and apoptosis, we focused on osteoclast‐osteoblast crosstalk coupled with S1P.^92^ Interestingly, FTY720 acts as an inhibitor of the S1P receptor and was tested in clinical experiments in 2017.^93^ In addition, S1P acts as a bridge linking macrophages with inflammatory osteolysis and bone metastasis, which provides us with a novel drug target. Thus, research on S1P and its receptors is of great academic and clinical value. However, we have little evidence to support our hypotheses, and more studies are needed to determine whether S1P exerts the same effects in bone as in other tissues.

## CONFLICT OF INTEREST

The authors declare no conflict of interest.

## AUTHOR CONTRIBUTIONS

Shiwu Dong and Yueqi Chen conceived the idea. Lincheng Zhang, Wenhui Hu and Yueqi Chen wrote and edited the manuscript. Lincheng Zhang, Yutong Dong and Yiran Wang drew the figures and organized the table. Shiwu Dong and Yueqi Chen approved the final version of the manuscript. All authors critically revised the manuscript.

## Data Availability

The data used to support the findings of this study are available from the corresponding author upon request.

## References

[1] Matsuo K , Irie N . Osteoclast‐osteoblast communication. Arch Biochem Biophys. 2008;473:201‐209. 18406338 10.1016/j.abb.2008.03.027

[2] Rosen H , Goetzl EJ . Sphingosine 1‐phosphate and its receptors: an autocrine and paracrine network. Nat Rev Immunol. 2005;5:560‐570. 15999095 10.1038/nri1650

[3] Sato K , Okajima F . Role of sphingosine 1‐phosphate in anti‐atherogenic actions of high‐density lipoprotein. World J Bio Chem. 2010;1:327‐337. 21537467 10.4331/wjbc.v1.i11.327 PMC3083937

[4] Takuwa Y . Subtype‐specific differential regulation of Rho family G proteins and cell migration by the Edg family sphingosine‐1‐phosphate receptors. Biochim Biophys Acta. 2002;1582:112‐120. 12069818 10.1016/s1388-1981(02)00145-2

[5] Fukuhara S , Simmons S , Kawamura S , et al. The sphingosine‐1‐phosphate transporter Spns2 expressed on endothelial cells regulates lymphocyte trafficking in mice. J Clin Invest. 2012;122:1416‐1426. 22406534 10.1172/JCI60746 PMC3314466

[6] Hait NC , Allegood J , Maceyka M , et al. Regulation of histone acetylation in the nucleus by sphingosine‐1‐phosphate. Science. 2009;325:1254‐1257. 19729656 10.1126/science.1176709 PMC2850596

[7] Meshcheryakova A , Mechtcheriakova D , Pietschmann P . Sphingosine 1‐phosphate signaling in bone remodeling: multifaceted roles and therapeutic potential. Expert Opin Ther Targets. 2017;21:725‐737. 28524744 10.1080/14728222.2017.1332180 PMC5470107

[8] Zhou J , Saba JD . Identification of the first mammalian sphingosine phosphate lyase gene and its functional expression in yeast. Biochem Biophys Res Commun. 1998;242:502‐507. 9464245 10.1006/bbrc.1997.7993

[9] van Veldhoven PP , Mannaerts GP . Sphingosine‐phosphate lyase. Adv Lipid Res. 1993;26:69‐98. 8379460

[10] Sartawi Z , Schipani E , Ryan KB , Waeber C . Sphingosine 1‐phosphate (S1P) signalling: role in bone biology and potential therapeutic target for bone repair. Pharmacol Res. 2017;125:232‐245. 28855094 10.1016/j.phrs.2017.08.013 PMC7253298

[11] Chen Y , Dou CE , Yi J , et al. Inhibitory effect of vanillin on RANKL‐induced osteoclast formation and function through activating mitochondrial‐dependent apoptosis signaling pathway. Life Sci. 2018;208:305‐314. 30055205 10.1016/j.lfs.2018.07.048

[12] Ishii M , Egen JG , Klauschen F , et al. Sphingosine‐1‐phosphate mobilizes osteoclast precursors and regulates bone homeostasis. Nature. 2009;458:524‐528. 19204730 10.1038/nature07713 PMC2785034

[13] Ishii M , Kikuta J . Sphingosine‐1‐phosphate signaling controlling osteoclasts and bone homeostasis. Biochim Biophys Acta. 2013;1831:223‐227. 22691949 10.1016/j.bbalip.2012.06.002

[14] Ishii M , Kikuta J , Shimazu Y , Meier‐Schellersheim M , Germain RN . Chemorepulsion by blood S1P regulates osteoclast precursor mobilization and bone remodeling in vivo. J Exp Med. 2010;207:2793‐2798. 21135136 10.1084/jem.20101474 PMC3005230

[15] Kono M , Belyantseva IA , Skoura A , et al. Deafness and stria vascularis defects in S1P2 receptor‐null mice. J Biol Chem. 2007;282:10690‐10696. 17284444 10.1074/jbc.M700370200

[16] Ryu J , Kim HJ , Chang E‐J , Huang H , Banno Y , Kim H‐H . Sphingosine 1‐phosphate as a regulator of osteoclast differentiation and osteoclast‐osteoblast coupling. EMBO J. 2006;25:5840‐5851. 17124500 10.1038/sj.emboj.7601430 PMC1698879

[17] Kikuta J , Kawamura S , Okiji F , et al. Sphingosine‐1‐phosphate‐mediated osteoclast precursor monocyte migration is a critical point of control in antibone‐resorptive action of active vitamin D. Proc Natl Acad Sci U S A. 2013;110:7009‐7013. 23569273 10.1073/pnas.1218799110 PMC3637769

[18] Matsuzaki E , Hiratsuka S , Hamachi T , et al. Sphingosine‐1‐phosphate promotes the nuclear translocation of beta‐catenin and thereby induces osteoprotegerin gene expression in osteoblast‐like cell lines. Bone. 2013;55:315‐324. 23612487 10.1016/j.bone.2013.04.008

[19] Kim B‐J , Shin K‐O , Kim H , et al. The effect of sphingosine‐1‐phosphate on bone metabolism in humans depends on its plasma/bone marrow gradient. J Endocrinol Invest. 2016;39:297‐303. 26219613 10.1007/s40618-015-0364-x

[20] Hsu L‐C , Reddy SV , Yilmaz Ö , Yu H . Sphingosine‐1‐phosphate receptor 2 controls podosome components induced by RANKL affecting osteoclastogenesis and bone resorption. Cells. 2019;8(1):17. 10.3390/cells8010017 PMC6357083 30609675

[21] Udagawa N , Takahashi N , Akatsu T , et al. Origin of osteoclasts: mature monocytes and macrophages are capable of differentiating into osteoclasts under a suiTable microenvironment prepared by bone marrow‐derived stromal cells. Proc Natl Acad Sci U S A. 1990;87:7260‐7264. 2169622 10.1073/pnas.87.18.7260 PMC54723

[22] Quint P , Ruan M , Pederson L , et al. Sphingosine 1‐phosphate (S1P) receptors 1 and 2 coordinately induce mesenchymal cell migration through S1P activation of complementary kinase pathways. J Bio Chem. 2013;288:5398‐5406. 23300082 10.1074/jbc.M112.413583 PMC3581421

[23] Okamoto H , Takuwa N , Yokomizo T , et al. Inhibitory regulation of Rac activation, membrane ruffling, and cell migration by the G protein‐coupled sphingosine‐1‐phosphate receptor EDG5 but not EDG1 or EDG3. Mol Cell Biol. 2000;20:9247‐9261. 11094076 10.1128/mcb.20.24.9247-9261.2000 PMC102182

[24] Lepley D , Paik JH , Hla T , et al. The G protein‐coupled receptor S1P2 regulates Rho/Rho kinase pathway to inhibit tumor cell migration. Cancer Res. 2005;65:3788‐3795. 15867375 10.1158/0008-5472.CAN-04-2311

[25] Sato C , Iwasaki T , Kitano S , Tsunemi S , Sano H . Sphingosine 1‐phosphate receptor activation enhances BMP‐2‐induced osteoblast differentiation. Biochem Biophys Res Commun. 2012;423:200‐205. 22659743 10.1016/j.bbrc.2012.05.130

[26] Higashi K , Matsuzaki E , Hashimoto Y , et al. Sphingosine‐1‐phosphate/S1PR2‐ mediated signaling triggers Smad1/5/8 phosphorylation and thereby induces Runx2 expression in osteoblasts. Bone. 2016;93:1‐11. 27612439 10.1016/j.bone.2016.09.003

[27] Vella G , Bergers G . Where have all the T cells gone? Immunity. 2018;49:592‐594. 30332627 10.1016/j.immuni.2018.10.006 PMC6589439

[28] Grey A , Xu X , Hill B , et al. Osteoblastic cells express phospholipid receptors and phosphatases and proliferate in response to sphingosine‐1‐phosphate. Calcif Tissue Int. 2004;74:542‐550. 15354862 10.1007/s00223-003-0155-9

[29] Grey A , Chen Q , Callon K , et al. The phospholipids sphingosine‐1‐phosphate and lysophosphatidic acid prevent apoptosis in osteoblastic cells via a signaling pathway involving G(i) proteins and phosphatidylinositol‐3 kinase. Endocrinology. 2002;143:4755‐4763. 12446603 10.1210/en.2002-220347

[30] Sukocheva OA . Expansion of sphingosine kinase and sphingosine‐1‐phosphate receptor function in normal and cancer cells: from membrane restructuring to mediation of estrogen signaling and stem cell programming. Int J Mol Sci. 2018;19:E420. 29385066 10.3390/ijms19020420 PMC5855642

[31] Lampasso JD , Kamer A , Margarone J , Dziak R . Sphingosine‐1‐phosphate effects on PKC isoform expression in human osteoblastic cells. Prostaglandins Leukot Essent Fatty Acids. 2001;65:139‐146. 11728164 10.1054/plef.2001.0302

[32] Hutami IR , Izawa T , Mino‐Oka A , et al. Fas/S1P1 crosstalk via NF‐kappaB activation in osteoclasts controls subchondral bone remodeling in murine TMJ arthritis. Biochem Biophys Res Commun. 2017;490:1274‐1281. 28687489 10.1016/j.bbrc.2017.07.006

[33] Hwang SY , Putney JW Jr . Calcium signaling in osteoclasts. Biochim Biophys Acta. 2011;1813:979‐983. 21075150 10.1016/j.bbamcr.2010.11.002 PMC3078988

[34] Wang H , Tian L , Liu J , et al. The osteogenic niche is a calcium reservoir of bone micrometastases and confers unexpected therapeutic vulnerability. Cancer Cell. 2018;34(5):823‐839.e7. 30423299 10.1016/j.ccell.2018.10.002 PMC6239211

[35] Duncan RL , Akanbi KA , Farach‐Carson MC . Calcium signals and calcium channels in osteoblastic cells. Semin Nephrol. 1998;18:178‐190. 9541272

[36] Wu X , Pan G , McKenna MA , Zayzafoon M , Xiong W‐C , McDonald JM . RANKL regulates Fas expression and Fas‐mediated apoptosis in osteoclasts. J Bone Miner Res. 2005;20:107‐116. 15619676 10.1359/JBMR.041022

[37] Dobrosak C , Gooi JH . Increased sphingosine‐1‐phosphate production in response to osteocyte mechanotransduction. Bone Rep. 2017;7:114‐120. 29085869 10.1016/j.bonr.2017.10.002 PMC5651498

[38] Ji F , Mao LI , Liu Y , et al. K6PC‐5, a novel sphingosine kinase 1 (SphK1) activator, alleviates dexamethasone‐induced damages to osteoblasts through activating SphK1‐Akt signaling. Biochem Biophys Res Commun. 2015;458:568‐575. 25680461 10.1016/j.bbrc.2015.02.007

[39] Brizuela L , Martin C , Jeannot P , et al. Osteoblast‐derived sphingosine 1‐phosphate to induce proliferation and confer resistance to therapeutics to bone metastasis‐derived prostate cancer cells. Mol Oncol. 2014;8:1181‐1195. 24768038 10.1016/j.molonc.2014.04.001 PMC5528572

[40] Kozawa O , Tokuda H , Matsuno H , Uematsu T . Activation of mitogen‐activated protein kinase is involved in sphingosine 1‐phosphate‐stimulated interleukin‐6 synthesis in osteoblasts. FEBS Lett. 1997;418:149‐151. 9414115 10.1016/s0014-5793(97)01366-5

[41] Coughlan T , Dockery F . Osteoporosis and fracture risk in older people. Clin Med. 2014;14:187‐191. 10.7861/clinmedicine.14-2-187 PMC4953292 24715132

[42] Jung J , Park JS , Righesso L , et al. Effects of an oral bisphosphonate and three intravenous bisphosphonates on several cell types in vitro. Clin Oral Investig. 2018;22:2527‐2534. 10.1007/s00784-018-2349-6 29388023

[43] Diel IJ , Bergner R , Grotz KA . Adverse effects of bisphosphonates: current issues. J Support Oncol. 2007;5:475‐482. 18240669

[44] Bone HG , Bolognese MA , Yuen CK , et al. Effects of denosumab treatment and discontinuation on bone mineral density and bone turnover markers in postmenopausal women with low bone mass. J Clin Endocrinol Metab. 2011;96:972‐980. 21289258 10.1210/jc.2010-1502

[45] Bandeira L , Lewiecki EM , Bilezikian JP . Romosozumab for the treatment of osteoporosis. Expert Opin Biol Ther. 2017;17:255‐263. 28064540 10.1080/14712598.2017.1280455

[46] Lemaire V , Cox DR . Dynamics of bone cell interactions and differential responses to PTH and antibody‐based therapies. Bull Math Biol. 2019;81(9):3575‐3622. 30460589 10.1007/s11538-018-0533-0

[47] Weske S , Vaidya M , Reese A , et al. Targeting sphingosine‐1‐phosphate lyase as an anabolic therapy for bone loss. Nat Med. 2018;24:667‐678. 29662200 10.1038/s41591-018-0005-y

[48] Keller J , Catala‐Lehnen P , Huebner AK , et al. Calcitonin controls bone formation by inhibiting the release of sphingosine 1‐phosphate from osteoclasts. Nat Commun. 2014;5:5215. 25333900 10.1038/ncomms6215 PMC4205484

[49] Tantikanlayaporn D , Tourkova IL , Larrouture Q , et al. Sphingosine‐1‐phosphate modulates the effect of estrogen in human osteoblasts. JBMR Plus. 2018;2:217‐226. 30123862 10.1002/jbm4.10037 PMC6095197

[50] Sukocheva O , Wadham C , Holmes A , et al. Estrogen transactivates EGFR via the sphingosine 1‐phosphate receptor Edg‐3: the role of sphingosine kinase‐1. J Cell Biol. 2006;173:301‐310. 16636149 10.1083/jcb.200506033 PMC2063820

[51] Sukocheva O , Wadham C , Xia P . Estrogen defines the dynamics and destination of transactivated EGF receptor in breast cancer cells: role of S1P₃ receptor and Cdc42. Exp Cell Res. 2013;319:455‐465. 23142484 10.1016/j.yexcr.2012.10.014

[52] Carpio LC , Shiau H , Dziak R . Changes in sphingolipid levels induced by epidermal growth factor in osteoblastic cells. Effects of these metabolites on cytosolic calcium levels. Prostaglandins Leukot Essent Fatty Acids. 2000;62:225‐232. 10882186 10.1054/plef.2000.0147

[53] Pederson L , Ruan M , Westendorf JJ , Khosla S , Oursler MJ . Regulation of bone formation by osteoclasts involves Wnt/BMP signaling and the chemokine sphingosine‐1‐phosphate. Proc Natl Acad Sci U S A. 2008;105:20764‐20769. 19075223 10.1073/pnas.0805133106 PMC2603259

[54] Lotinun S , Kiviranta R , Matsubara T , et al. Osteoclast‐specific cathepsin K deletion stimulates S1P‐dependent bone formation. J Clin Invest. 2013;123:666‐681. 23321671 10.1172/JCI64840 PMC3561821

[55] Hutami IR , Tanaka E , Izawa T . Crosstalk between Fas and S1P1 signaling via NF‐kB in osteoclasts controls bone destruction in the TMJ due to rheumatoid arthritis. Jpn Dent Sci Rev. 2019;55:12‐19. 30733840 10.1016/j.jdsr.2018.09.004 PMC6354287

[56] Hou GQ , Guo C , Song GH , et al. Lipopolysaccharide (LPS) promotes osteoclast differentiation and activation by enhancing the MAPK pathway and COX‐2 expression in RAW264.7 cells. Int J Mol Sci. 2013;32:503‐510. 10.3892/ijmm.2013.1406 23740407

[57] Xiao L , Zhou Y , Zhu L , et al. SPHK1‐S1PR1‐RANKL Axis Regulates the Interactions Between Macrophages and BMSCs in Inflammatory Bone Loss. J Bone Miner Res. 2018;33:1090‐1104. 29377379 10.1002/jbmr.3396

[58] Huang R , Wang X , Zhou Y , Xiao Y . RANKL‐induced M1 macrophages are involved in bone formation. Bone Res. 2017;5:17019. 29263936 10.1038/boneres.2017.19 PMC5645773

[59] Lee H , Liao JJ , Graeler M , et al. Lysophospholipid regulation of mononuclear phagocytes. Biochim Biophys Acta. 2002;1582:175‐177. 12069826 10.1016/s1388-1981(02)00153-1

[60] Pettus BJ , Bielawski J , Porcelli AM , et al. The sphingosine kinase 1/sphingosine‐1‐phosphate pathway mediates COX‐2 induction and PGE2 production in response to TNF‐alpha. FASEB J. 2003;17:1411‐1421. 12890694 10.1096/fj.02-1038com

[61] Müller J , von Bernstorff W , Heidecke C‐D , Schulze T . Differential S1P receptor profiles on M1‐ and M2‐polarized macrophages affect macrophage cytokine production and migration. Biomed Res Int. 2017;2017:7584621. 28367448 10.1155/2017/7584621 PMC5358463

[62] Hammad SM , Crellin HG , Wu BX , Melton J , Anelli V , Obeid LM . Dual and distinct roles for sphingosine kinase 1 and sphingosine 1 phosphate in the response to inflammatory stimuli in RAW macrophages. Prostaglandins Other Lipid Mediat. 2008;85:107‐114. 18166496 10.1016/j.prostaglandins.2007.11.002 PMC2290737

[63] Heo JY , Im DS . Pro‐Inflammatory role of S1P3 in macrophages. Biomol Ther (Seoul). 2019;27:373‐380. 30917625 10.4062/biomolther.2018.215 PMC6609111

[64] Keul P , Lucke S , von Wnuck Lipinski K , et al. Sphingosine‐1‐phosphate receptor 3 promotes recruitment of monocyte/macrophages in inflammation and atherosclerosis. Circ Res. 2011;108:314‐323. 21164103 10.1161/CIRCRESAHA.110.235028

[65] Yang J , Yang L , Tian L , et al. Sphingosine 1‐phosphate (S1P)/S1P receptor2/3 axis promotes inflammatory M1 polarization of bone marrow‐derived monocyte/macrophage via G(alpha)i/o/PI3K/JNK pathway. Cell Physiol Biochem. 2018;49:1677‐1693. 30231248 10.1159/000493611

[66] Hughes JE , Srinivasan S , Lynch KR , Proia RL , Ferdek P , Hedrick CC . Sphingosine‐1‐phosphate induces an antiinflammatory phenotype in macrophages. Cir Res. 2008;102:950‐958. 10.1161/CIRCRESAHA.107.170779 PMC2875063 18323526

[67] Dou CE , Ding N , Zhao C , et al. Estrogen deficiency‐mediated M2 macrophage osteoclastogenesis contributes to M1/M2 ratio alteration in ovariectomized osteoporotic mice. J Bone Miner Res. 2018;33:899‐908. 29281118 10.1002/jbmr.3364

[68] Zhao S , Adebiyi MG , Zhang Y , et al. Sphingosine‐1‐phosphate receptor 1 mediates elevated IL‐6 signaling to promote chronic inflammation and multitissue damage in sickle cell disease. FASEB J. 2018;32:2855‐2865. 29401601 10.1096/fj.201600788RR PMC5901384

[69] Michaud J , Im DS , Hla T . Inhibitory role of sphingosine 1‐phosphate receptor 2 in macrophage recruitment during inflammation. J Immunol. 2010;184:1475‐1483. 20042570 10.4049/jimmunol.0901586 PMC3068864

[70] Tanaka K , Hashizume M , Mihara M , Yoshida H , Suzuki M , Matsumoto Y . Anti‐interleukin‐6 receptor antibody prevents systemic bone mass loss via reducing the number of osteoclast precursors in bone marrow in a collagen‐induced arthritis model. Clin Exp Immunol. 2014;175:172‐180. 24028747 10.1111/cei.12201 PMC3892408

[71] Allende ML , Bektas M , Lee BG , et al. Sphingosine‐1‐phosphate lyase deficiency produces a pro‐inflammatory response while impairing neutrophil trafficking. J Biol Chem. 2011;286:7348‐7358. 21173151 10.1074/jbc.M110.171819 PMC3044991

[72] Aoki M , Aoki H , Ramanathan R , et al. Sphingosine‐1‐phosphate signaling in immune cells and inflammation: roles and therapeutic potential. Mediators Inflamm. 2016;2016:8606878. 26966342 10.1155/2016/8606878 PMC4761394

[73] Baeyens A , Fang V , Chen C , Schwab SR . Exit strategies: S1P signaling and T cell migration. Trends Immunol. 2015;36:778‐787. 26596799 10.1016/j.it.2015.10.005 PMC4832571

[74] Zheng Z , Zeng YZ , Ren K , et al. S1P promotes inflammation‐induced tube formation by HLECs via the S1PR1/NF‐kappaB pathway. Int Immunopharmacol. 2019;66:224‐235. 30476824 10.1016/j.intimp.2018.11.032

[75] Davila D , Antoniou A , Chaudhry MA . Evaluation of osseous metastasis in bone scintigraphy. Semin Nucl Med. 2015;45:3‐15. 25475375 10.1053/j.semnuclmed.2014.07.004

[76] Gude DR , Alvarez SE , Paugh SW , et al. Apoptosis induces expression of sphingosine kinase 1 to release sphingosine‐1‐phosphate as a "come‐and‐get‐me" signal. FASEB J. 2008;22:2629‐2638. 18362204 10.1096/fj.08-107169 PMC2493451

[77] Ley S , Weigert A , Weichand B , et al. The role of TRKA signaling in IL‐10 production by apoptotic tumor cell‐activated macrophages. Oncogene. 2013;32:631‐640. 22410777 10.1038/onc.2012.77

[78] Weigert A , Tzieply N , von Knethen A , et al. Tumor cell apoptosis polarizes macrophages role of sphingosine‐1‐phosphate. Mol Biol Cell. 2007;18:3810‐3819. 17652460 10.1091/mbc.E06-12-1096 PMC1995721

[79] Rodriguez YI , Campos LE , Castro MG , Aladhami A , Oskeritzian CA , Alvarez SE . Sphingosine‐1 phosphate: a new modulator of immune plasticity in the tumor microenvironment. Front Oncol. 2016;6:218. 27800303 10.3389/fonc.2016.00218 PMC5066089

[80] Brecht K , Weigert A , Hu J , et al. Macrophages programmed by apoptotic cells promote angiogenesis via prostaglandin E2. FASEB J. 2011;25:2408‐2417. 21450910 10.1096/fj.10-179473

[81] Rathinasamy A , Domschke C , Ge Y , et al. Tumor specific regulatory T cells in the bone marrow of breast cancer patients selectively upregulate the emigration receptor S1P1. Cancer Immunol Immunother. 2017;66:593‐603. 28224210 10.1007/s00262-017-1964-4 PMC5406429

[82] van der Weyden L , Arends MJ , Campbell AD , et al. Genome‐wide in vivo screen identifies novel host regulators of metastatic colonization. Nature. 2017;541:233‐236. 28052056 10.1038/nature20792 PMC5603286

[83] Ponnusamy S , Selvam SP , Mehrotra S , et al. Communication between host organism and cancer cells is transduced by systemic sphingosine kinase 1/sphingosine 1‐phosphate signalling to regulate tumour metastasis. EMBO Mol Med. 2012;4:761‐775. 22707406 10.1002/emmm.201200244 PMC3494075

[84] Jung M , Ören B , Mora J , et al. Lipocalin 2 from macrophages stimulated by tumor cell‐derived sphingosine 1‐phosphate promotes lymphangiogenesis and tumor metastasis. Sci Signal. 2016;9:ra64. 27353364 10.1126/scisignal.aaf3241

[85] Miyazaki Y , Niino M , Kanazawa I , et al. Fingolimod suppresses bone resorption in female patients with multiple sclerosis. J Neuroimmunol. 2016;298:24‐31. 27609272 10.1016/j.jneuroim.2016.06.007

[86] Grenald SA , Doyle TM , Zhang H , et al. Targeting the S1P/S1PR1 axis mitigates cancer‐induced bone pain and neuroinflammation. Pain. 2017;158:1733‐1742. 28570482 10.1097/j.pain.0000000000000965 PMC5580091

[87] D'Ambrosio D , Freedman MS , Prinz J . Ponesimod, a selective S1P1 receptor modulator: a potential treatment for multiple sclerosis and other immune‐mediated diseases. Ther Adv Chronic Dis. 2016;7:18‐33. 26770667 10.1177/2040622315617354 PMC4707431

[88] Hundehege P , Cerina M , Eichler S , et al. The next‐generation sphingosine‐1 receptor modulator BAF312 (siponimod) improves cortical network functionality in focal autoimmune encephalomyelitis. Neural Regen Res. 2019;14:1950‐1960. 31290453 10.4103/1673-5374.259622 PMC6676873

[89] Scott FL , Clemons B , Brooks J , et al. Ozanimod (RPC1063) is a potent sphingosine‐1‐phosphate receptor‐1 (S1P1) and receptor‐5 (S1P5) agonist with autoimmune disease‐modifying activity. Br J Pharmacol. 2016;173:1778‐1792. 26990079 10.1111/bph.13476 PMC4867749

[90] El Jamal A , Bougault C , Mebarek S , Magne D , Cuvillier O , Brizuela L . The role of sphingosine 1‐phosphate metabolism in bone and joint pathologies and ectopic calcification. Bone. 2020;130:115087. 31648078 10.1016/j.bone.2019.115087

[91] Xiao L , Zhou Y , Friis T , Beagley K , Xiao Y . S1P–S1PR1 signaling: the "Sphinx" in osteoimmunology. Front Immunol. 2019;10:1409. 31293578 10.3389/fimmu.2019.01409 PMC6603153

[92] Ng ML , Yarla NS , Menschikowski M , Sukocheva OA . Regulatory role of sphingosine kinase and sphingosine‐1‐phosphate receptor signaling in progenitor/stem cells. World J Stem Cells. 2018;10(9):119‐133. 30310531 10.4252/wjsc.v10.i9.119 PMC6177561

[93] Beider K , Rosenberg E , Bitner H , et al. The sphingosine‐1‐phosphate modulator FTY720 targets multiple myeloma via the CXCR4/CXCL12 pathway. Clin Cancer Res. 2017;23:1733‐1747. 27697999 10.1158/1078-0432.CCR-15-2618

